# Inactivation of human coronaviruses using an automated room disinfection device

**DOI:** 10.1038/s41598-023-47082-z

**Published:** 2023-11-16

**Authors:** Nicholas A. Lundquist, Legesse G. Kifelew, Sait Elmas, Zhongfan Jia, Peter G. Speck, Justin M. Chalker

**Affiliations:** 1 Institute for Nanoscale Science and Technology, College of Science and Engineering, Flinders University, Bedford Park, SA 5042 Australia; 2 Molecular Biosciences, College of Science and Engineering, Flinders University, Bedford Park, SA 5042 Australia

**Keywords:** SARS-CoV-2, Electrocatalysis

## Abstract

The emergence of more virulent and epidemic strains of viruses, especially in the context of COVID-19, makes it more important than ever to improve methods of decontamination. The objective of this study was to evaluate the potential of on-demand production of chlorine species to inactivate human coronaviruses. The commercial prototype disinfection unit was provided by Unipolar Water Technologies. The Unipolar device generates active chlorine species using an electrochemical reaction and dispenses the disinfectant vapour onto surfaces with an aspirator. The minimum effective concentration and exposure time of disinfectant were evaluated on human hepatoma (Huh7) cells using 50% tissue culture infectious dose (TCID_50_) assay and human coronavirus 229E (HCoV-229E), a surrogate for pathogenic human coronaviruses. We showed that chlorine species generated in the Unipolar device inactivate HCoV-229E on glass surfaces at ≥ 400 parts per million active chlorine concentration with a 5 min exposure time. Here, inactivation refers to the inability of the virus to infect the Huh7 cells. Importantly, no toxic effect was observed on Huh7 cells for any of the active chlorine concentrations and contact times tested.

## Introduction

**Figure 1:**
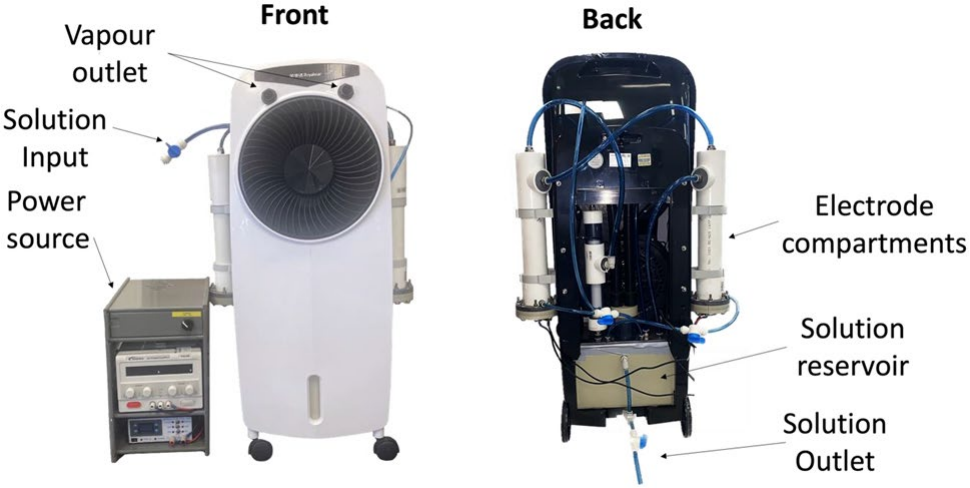
Images of the Unipolar ARD device, with key components labelled.

The emergence of more virulent and epidemic strains of virus, especially in light of the recent COVID-19 pandemic, makes it important to improve methods of decontamination^1,2^. Automated room disinfection (ARD) systems are used to sterilise contaminated surfaces with minimal assistance from human operators and have been developed to improve both the efficacy and reliability of hospital disinfection^3–7^. Vapour-generating ARD systems are important as they limit human error and generate fine mists that can cover, penetrate and disinfect complex surfaces^8^. This study investigated the use of the Unipolar Water Technologies ARD system for its effectiveness in the disinfection of the SARS-CoV-2 surrogate, HCoV-229E. The Unipolar Water Technologies ARD system operates by electrochemical generation of active chlorine species such as aqueous hypochlorous acid which are then dispensed in vapour form with an aspirator (Fig. 1).

Vaccines are a powerful measure to protect human health against pathogenic viruses, yet there is a significant challenge in achieving global population immunization against highly pathogenic coronavirus diseases because of vaccine efficacy, logistics, and availability problems^9^. Anti-viral treatments are not always fully effective^10,11^. In the absence of enough vaccines and effective therapeutic agents, protective measures such as mask-wearing^12^ and viral disinfection are important^13^. Human coronaviruses such as SARS-CoV-2 and HCoV-229E are transmitted through exposure to a cough or sneeze, inhalation of aerosolized virus, and through contact of contaminated surfaces^14–16^. The persistence of human coronaviruses on inanimate surfaces varies from a few minutes to days^14,17–19^ and contact between contaminated surfaces and hands with mucus membranes is a source of indirect transmission for coronaviruses^17^ and other viruses such as Ebolaviruses^20^. Effective surface disinfection may help ensure early containment and prevent further viral spread^21^. For these reasons, there has been a growing interest in developing more effective surface decontamination methods^22^ and improving the standard of disinfection to eliminate the increased risk of infection^23–25^. The minimum effective concentration and exposure time required for action of a disinfectant are the main factors in assessing its effectiveness against coronavirus and surrogate viruses^26^. HCoV-229E and murine hepatitis viruses are recommended viruses to study the efficacy of a disinfectant on hard surfaces and medical devices^27,28^. Huh7 cell lines are preferred by many researchers for these studies in assessing viral infection^29,30^, with the 50% tissue culture infectious dose assay used to quantify virus titre, infectivity, and effectiveness of the disinfectant^31^. The 50% tissue culture infectious dose value represents the number of viral particles needed to produce cytopathic effects in 50% of the cell culture or wells containing the treated cell culture after a defined period^32^.

Previous studies have shown that vapour-generating ARD systems provide an effective means to disinfect large spaces such as hospitals, nursing homes and quarantine facilities^33^. ARD systems have been demonstrated as effective means to decontaminate rooms^34,35^, multibed bays^34,36,37^ and entire units^34,38,39^ during outbreaks of *Clostridioides difficile*^40^, *Acinetobacter baumannii*^41,42^, methicillin-resistant *Staphylococcus aureus* and methicillin‐susceptible *S. aureus*^36,39,43^, multidrug‐resistant Gram‐negative bacteria^37,38,44^, and viruses including Severe Acute Respiratory Syndrome Coronavirus 2 (SARS-CoV-2) and Lassa virus^45–47^. They have been demonstrated to successfully mitigate the risk posed from prior room occupancy^24,34,48–50^. These systems have recently been investigated for their potential to combat COVID-19^51^. Although these systems typically use H_2_O_2_, a recent report suggested that the use of cold vapour from a 1% sodium hypochlorite solution is more effective than other disinfection strategies being used to inactivate SARS-Cov-2^8^. The World Health Organization also recommends the use of chlorinated disinfectant solutions^52^, which are high-efficacy disinfectants with a low chlorine concentration^27^.

This study aimed to evaluate the effect of an active chlorine-containing vapour on HCoV-229E. The ARD device tested generates active chlorine solutions on demand, and then dispenses the disinfectant vapour using an aspirator. Key objectives of the study were to characterize the disinfectant produced in this device, understand how vapour generation influences active chlorine concentration, and establish the exposure time required for inactivation of the SARS-CoV-2 surrogate, HCoV-229E.

## Materials and methods

### Active chlorine concentration analysis

An aqueous solution of methyl orange (14 ppm) and potassium bromide (100 ppm) was prepared, and all testing was carried out in open air at 20 °C. Five mL of this solution was transferred into a 20 mL glass vial. To this solution, 0.5 mL of the disinfectant solution was added. The absorbance of the sample at 469 nm was then measured using a spectrophotometer. If the absorbance was below 0.1 (indicating complete consumption of the methyl orange by reaction with active chlorine), the experiment was repeated with a more dilute sample. The absorbance at 469 nm was then used to determine the concentration of active chlorine based on a calibration curve. An equimolar reaction between methyl orange and active chlorine species is assumed in this assay.

### Automated room disinfectant (ARD) system

The ARD system used in this study was provided by Unipolar Water Technologies^53^. This device generates active chlorine species using electrolysis of aqueous solutions of sodium chloride. The Unipolar ARD system then converts the bulk disinfectant solution into a vapour that is dispersed onto a target. The active chlorine concentration produced by this device can be altered by changing the initial NaCl_(aq)_ concentration, the applied current or the run time. The device is filled with 5.5 L of NaCl_(aq)_ solution of a desired concentration, and all sampling can be done through the outlet valve in the main reservoir at the bottom of the device (Fig. 1).

### Virus propagation

Viral propagation and application of active chlorine treatment were adapted from protocols previously described^54,55^. Briefly: 1 mL Huh7 suspension (1.3 × 10^6^ cells/mL) was mixed with 8 mL Dulbecco's Modified Eagle Medium (DMEM) and 1 mL foetal bovine serum (FBS). The mixture was incubated at 37 °C with 5% CO_2_ in a sterile 75 mL cell culture flask. When ≥ 90% confluent cell growth was observed, the Huh7 culture was infected by HCoV-229E (3 × 10^6^ TCID_50_ units/mL) and incubated for 5 days or until ≥ 90% of the culture showed cytopathic effect. Then, serum-free DMEM was added to the culture flask, and cells were harvested using a cell scraper and shaking. The suspension was centrifuged at 1000×*g* for 15 min. The pelleted cells were collected with serum-free DMEM and subjected to three rapid freeze–thaw cycles. The cell lysates were collected, mixed with 3 mL supernatant, aliquoted, and stored at −150 °C as crude virus samples; unpurified virus samples that may contain Huh7 host cells. Virus purification was carried out using the high purity™ lentivirus concentration kit (Creative Biogene, 45-1 Ramsay Road, Shirley, NY 11967, USA) following the manufacturer’s instructions. Virus stocks were quantified using 50% tissue culture infectious dose before use.

### Treatment with active chlorine disinfectant (general procedure)

**Figure 2:**
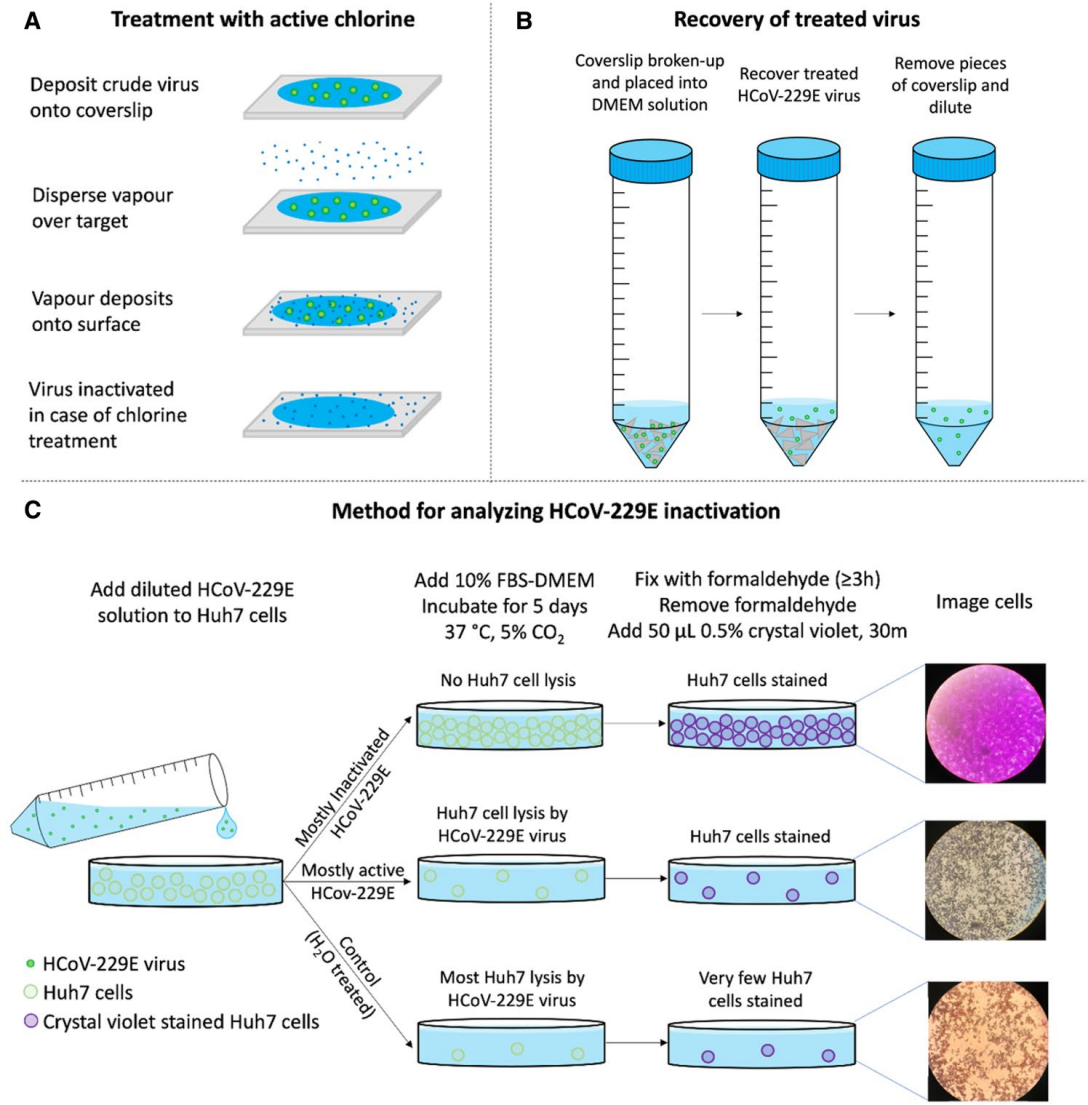
Images depicting the process of (**A**) treatment of HCoV-229E virus with active chlorine, (**B**) Recovery of the treated cells in solution, and (**C**) The method for analysing the amount of HCoV-229E inactivation.

The Unipolar disinfectant vapour was applied using a method adapted from similar carrier methods^54,56–58^. A carrier method is a disinfection effect measurement test in which microorganisms are applied to the surface of a carrier (stainless steel, glass or polyvinyl chloride), dried, and then treated with a disinfectant^59^. Carrier methods more accurately reflect the applications in real-life clinical practice^54^. In summary: 35 µL of HCoV-229E crude virus sample (3 × 10^6^ TCID_50_ units/mL) were put at the centre of a sterile 22 × 22 mm coverslip using a micropipette, gently spread all over the coverslip by the pipette tip and left at room temperature in a biosafety cabinet. Five minutes later, the coverslip containing the virus was transferred out of the biosafety cabinet and onto a glass stage placed on a bench within the lab, 18 cm away from the outlet of the Unipolar ARD device. Application of the disinfectant vapour (prepared at the specified concentration) onto the sample was then performed for varying amounts of time (referred to here as *treatment time*, ranging from 5 to 15 min). Note that in this case the treatment space is the volume of the room the samples are located within, however this experiment was also carried out by confining the treatment space into a smaller area (12,675 cm^3^). After application of the disinfectant mist, the sample was incubated further for varying amounts of time (referred to here as *exposure time,* ranging from 0 to 30 min). Following the exposure time, the coverslip was broken into smaller pieces and put in a 50 mL polypropylene tube containing 665 µL DMEM. Virus samples on the coverslip were suspended in the DMEM with careful shaking. Virus recovery was also made from sterile water-treated virus samples (treatment control groups) using the same procedure. Simultaneously, the effect of the disinfectant on the human cell line was checked. Accordingly, a coverslip in which only disinfectant vapour was applied with the same procedure was used to check the toxicity of the disinfectant on Huh7 cells. Toxicity was measured through the absence, presence, or degree of cytopathic effect. Ten-fold dilutions were made from each suspension in a 50 mL polypropylene tube and applied to each group of Huh7 cell cultures in 96-well tissue culture plates. Fifty microlitres of the suspension from each dilution was applied to at least eight wells containing ≥ 90% confluent Huh7 cell cultures and left on a rocking platform for an hour at room temperature. Then, 100 µL DMEM containing 10% foetal bovine serum was added to each well. Cultures treated with 50 µL of 10% foetal bovine serum—DMEM (FBS-DMEM) only were also used as the cell culture control group. The plates were then incubated for 5 days at 37 °C with 5% CO_2_. On day 6, the cultures were fixed with 100 µL of formaldehyde per well for ≥ 3 h. The formaldehyde was discarded, and cultures were stained with 50 µL 0.5% crystal violet per well for 30 min. Excess crystal violet was washed off using distilled water, and plates were air-dried and examined for cytopathic effects using an inverted microscope. Experiments were repeated three times with a minimum of three replicas each time unless indicated, and the mean is taken as a final result. This process is summarised in Fig. 2.

### Determination of minimum effective concentration of disinfectant vapour

To determine the minimum effective disinfectant concentration, the general procedure described above was adopted and varied in the disinfectant concentration. First, 35 µL of HCoV-229E virus at 3 × 10^6^ TCID_50_ units/mL was smeared on sterile 22 × 22 mm coverslips and left for five minutes at room temperature in a biosafety cabinet as above^54,56–58^. The coverslip containing the virus was transferred out of the biosafety cabinet and onto a glass stage placed on a bench in the lab before being contained in a smaller space by placing a large container with an opening on one side over the samples. The smeared virus samples were then treated with the disinfectant vapour with active chlorine concentrations of 200, 400, or 1000 parts per million (ppm) or sterile water for 10 min of treatment time using the Unipolar ARD at an 18 cm distance from the tip of the device’s nozzle to the target. After treatment, the samples were left at room temperature in a biosafety cabinet for an exposure time of an additional 10 min before they were processed as above. This experiment was repeated three times with three replicas each time.

### Determination of minimum effective exposure time of disinfectant vapour

To determine the minimum exposure time for the Unipolar disinfectant vapour to inactivate HCoV-229E virus, the general procedure described above was adopted and varied in exposure time for a disinfectant concentration of 400 ppm active chlorine. Accordingly, 35 µL of HCoV-229E at 3 × 10^6^ TCID_50_ units/mL concentration smeared on sterile 22 × 22 mm coverslips was left in a biosafety cabinet for 5 min at room temperature, in accordance with published protocols^54,56–58^. The coverslip containing the virus was transferred out of the biosafety cabinet and onto a glass stage on a bench in the lab before being contained in a smaller space by placing a large square plastic container (12,675 cm^3^) with an opening on one side over the samples. The virus samples were then treated with the disinfectant vapour containing 400 ppm active chlorine or sterile water (negative control) for treatment times of 10 min, after which they were transferred into the biosafety cabinet and left at room temperature for exposure times of 0-, 2-, 5-, 10-, 20-, and 30-min. At the end of each exposure time, samples were collected in DMEM and processed as described above.

## Results and discussion

### Active chlorine quantification method

**Figure 3:**
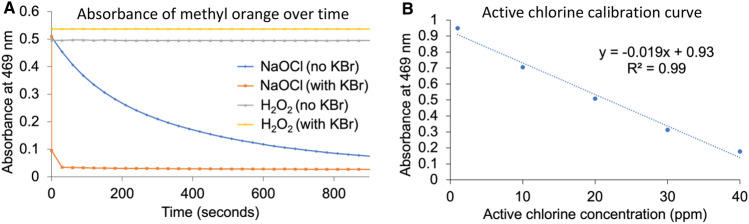
(**A**) UV absorbance at 469 nm for solutions of methyl orange treated with NaOCl and H_2_O_2_ with and without KBr present, (**B**) Calibration curve for active chlorine concentration based on the UV/Vis absorbance of methyl orange dye.

To quantitatively monitor the active chlorine generated by the Unipolar ARD, a rapid, dye-based method was developed. Treatment of methyl orange dye with the Unipolar disinfectant solution or vapour results in decolouration that can be quantified using UV/Vis spectroscopy. The absorbance was monitored at the isosbestic point (469 nm) for methyl orange to control for its pH-dependent absorbance^60,61^. Degradation of methyl orange using Unipolar disinfectant solution or vapour requires longer than 30 min to reach completion, however this reaction can be accelerated by the addition of KBr (100 ppm) (Fig. 3A). The KBr reacts with active chlorine species to generate more reactive hypobromous acid in situ^62^. The presence of KBr did not influence the UV/Vis absorbance at 469 nm, allowing for the instantaneous determination of active chlorine concentration. It was also important to note that this method was selective for active chlorine and was unaffected by the presence of H_2_O_2_. Using this strategy, a calibration curve was created through the addition of 0.5 mL of various concentrations of disinfectant solution to 5.0 mL of an aqueous solution containing 14.3 ppm methyl orange and 100 ppm KBr and recording the absorbance at 469 nm (Fig. 3B).

### Active chlorine quantification of the Unipolar ARD device

**Figure 4:**
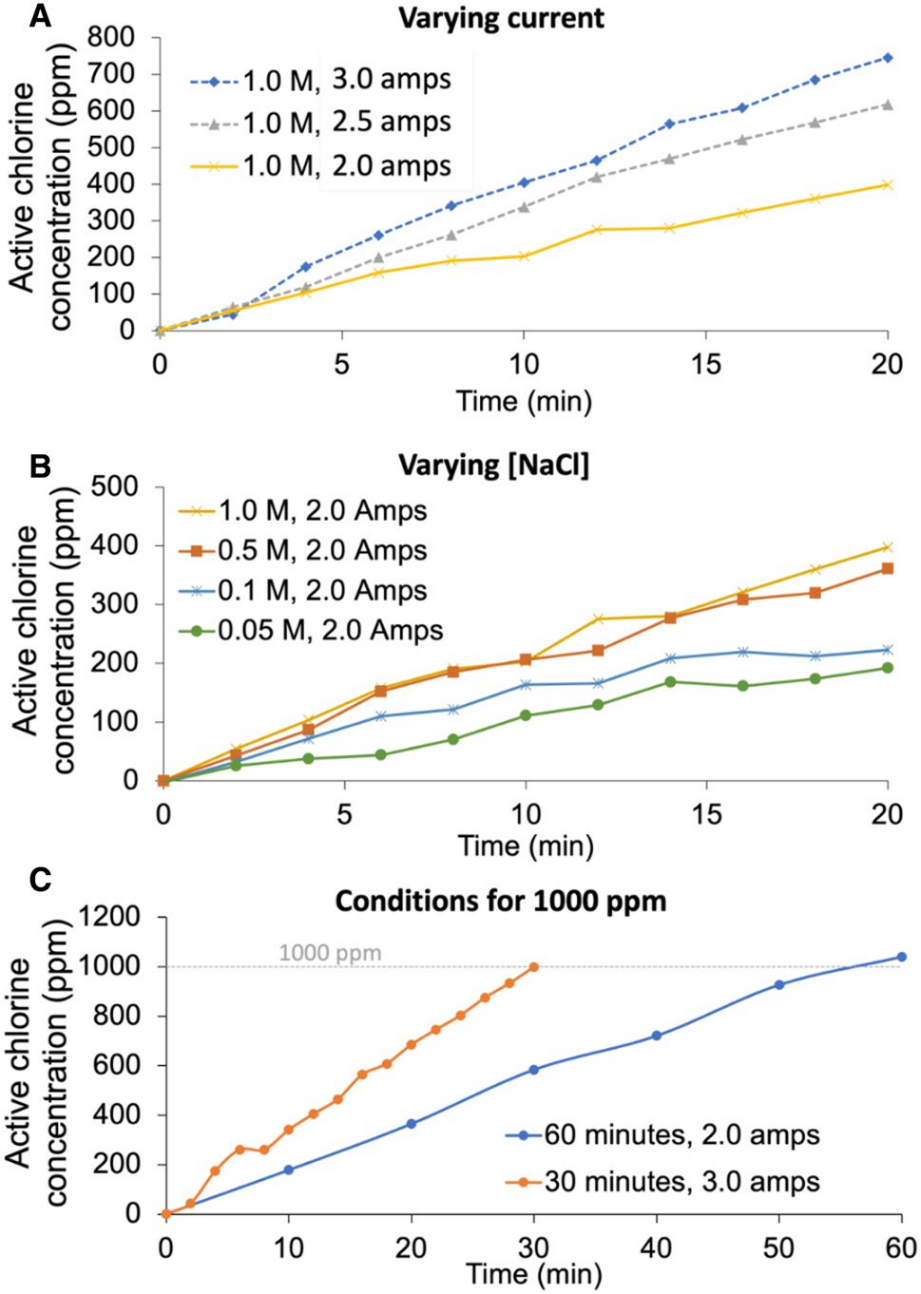
(**A**) Active chlorine production over electrolysis time varying the electrolyser amperage, (**B**) Active chlorine production over electrolysis time varying the initial NaCl_(aq)_ concentrations (**C**) Two sets of conditions used to produce 1000 ppm active chlorine for disinfectant experiments using initial NaCl(aq) concentrations of 1.0 M.

The Unipolar ARD device was used to produce active chlorine-containing disinfectant under varying sodium chloride concentrations and current. The active chlorine production in the solution reservoir was monitored over time using the methyl orange dye assay described above. Increasing the NaCl_(aq)_ concentration or the applied current increased the rate of active chlorine production as expected (Fig. 4A, B). Using this information, it was possible to tailor the run-time and NaCl_(aq)_ concentration of the initial solution to achieve a final solution with a desired active chlorine concentration of up to approximately 400 ppm in 20 min. To achieve concentrations of active chlorine up to 1000 ppm using an initial NaCl_(aq)_ concentration of 1.0 M, the run time was increased to approximately 1 h at 2.0 A or 30 min when the amperage was increased to 3.0 A (Fig. 4C). This control is useful for ARD devices so that the disinfectant concentration can be tuned as required.

**Figure 5:**
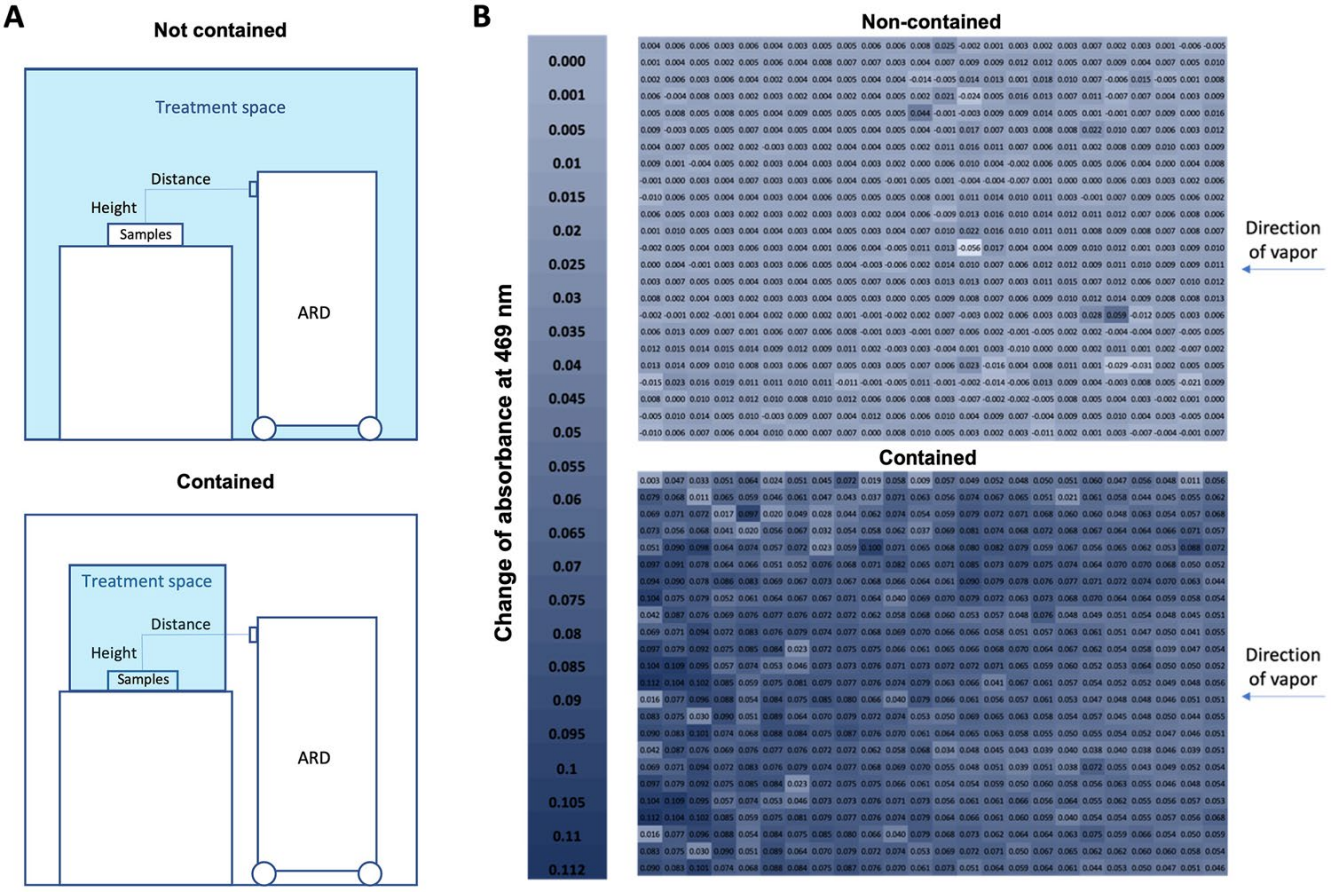
(**A**) Diagram showing the difference in treatment space when the sample is contained (bottom) versus when it is not contained (top). (**B**) Distribution of active chlorine across the surface after treatment when samples were contained (bottom) vs. uncontained (top). The confinement reduces the space that the disinfectant can travel. Values are for the change in absorbance at 469 nm in the 6 × 96 well plates. A larger change in absorbance (a reduction in absorbance) was observed in the confined treatment space, indicating a more efficient reaction of the active chlorine with the dye than observed for the non-confined space.

After electrolysis was complete, approximately 6 min were required to equilibrate the active chlorine concentration across the Unipolar ARD system. This equilibration is achieved by circulating the solution through the reservoir and electrode chambers with a pump operating at a flow rate of 4.5 L/min (Fig. 1). With this data and the concentration studies summarised in Fig. 2, the concentration of active chlorine in the disinfectant solution could be controlled. With that said, when this solution is converted into a vapour with the Unipolar aspirator, some of the active chlorine is lost due to volatilisation of chlorine species^63–65^. The active chlorine concentration in the reservoir is therefore the maximum active chlorine concentration in the resulting vapour. The effects of the distance and height from the sample as well as the size of the space the sample is contained within, on the distribution of the disinfectant were investigated. The treatment space and the setup of the experiment is described in Fig. 5A. Accordingly, an aqueous solution containing 14.3 ppm methyl orange and 100 ppm KBr was added to each well of 6 × 96 well plates. The plates were arranged in two rows of three with the long sides of each column next to each other. The Unipolar ARD was then placed in front of the plates and the vapour was dispersed across the sample area. The UV/Vis absorbance at 469 nm was then recorded for each well and the relative absorbance values were plotted as a colour map shown in Fig. 5. The distribution was uniform regardless of whether the device was placed at different heights and distances relative to the samples. The major difference observed occurred due to reducing the volume of the treated space (Fig. 5B). By reducing the volume of the treatment space, a higher rate of reaction (and disinfection when applied to virus samples) was observed across the samples. This demonstrates how efficiency of the surface treatment is proportional to the size of the space the vapour is dispersed within.

### Testing the unipolar ARD device for HCoV-229E virus inactivation

#### Effect of treatment space on HCoV-229E disinfection efficiency

To test the efficacy of the Unipolar ARD device against coronaviruses on hard surfaces, HCoV-229E was used as a model virus^27^. Based on a recently published European standard, titre reduction of coronaviruses due to effective disinfection must be at least 10,000-fold^54,66^. Virucidal activity of the chlorine-based disinfectant in this study was determined by the difference in logarithmic titre of the virus between control and treatment groups, referred to as reduction factor. A reduction factor of ≥ 4 was regarded as evidence of sufficient virucidal activity^21,56^. Initially, experiments were performed using a reservoir concentration of 400 ppm active chlorine, with exposure times of 1, 5 and 15 min, but minimal virus inactivation was observed in the larger treatment space. Therefore, the 15-min exposure time was then repeated but this time the samples were confined in a smaller treatment space (12,675 cm^3^). These initial experiments confirmed the effect of the size of the treatment space on treatment efficiency observed in dye experiments with virus inactivation only observed when the surface contaminated with the virus was confined in a smaller treatment space. Using a reservoir concentration of 400 ppm active chlorine, 100-fold inactivation of crude HCoV-229E was achieved in a confined space, whereas without space confinement, the level of inactivation was not significant. Under these conditions, the disinfectant vapour was also not toxic to Huh7 cells. These results were encouraging and prompted further tests to evaluate the effects of active chlorine concentration and exposure time on HCoV-229E inactivation efficiency. All the following experiments were performed in a confined space to ensure factors such as airflow and ventilation could be held constant between tests.

#### Minimum effective concentration of active chlorine for HCoV-229E disinfection

Next, treatment and exposure times were held at 10 min and the effect of active chlorine concentration was investigated. First, disinfectant vapour was generated from the Unipolar reservoir containing concentrations of 200, 400 and 1000 ppm active chlorine. Each concentration was evaluated for its effectiveness towards inactivation of HCoV-229E virus on the surface of a carrier material. The results revealed that both 400 and 1000 ppm active chlorine showed effective (> 4 log_10_) inactivation of HCoV-229E virus on carrier whereas insignificant (1.2 log_10_) virus inactivation occurred at 200 ppm active chlorine (Table 1).

**Table 1:** Concentration of HCoV-229E virus cells recovered after treatment with active chlorine disinfectant at various concentrations and exposure periods.

Active chlorine (ppm)	Exposure time (min)	HCoV-229E recovered (TCID_50_ units in log_10_/mL)	
		Active chlorine treated	Water treated (negative control)
200	0	4.3	5.3
	10	2.9	4.1
400	0	3	5
	10	0	5.6
1000	0	0	5.4
	10	0	4.8

Based on these results, 400 ppm active chlorine was chosen as the concentration to be used for further testing. It should be noted that testing virucidal activity of disinfectants using a carrier method imitates real-world conditions encountered in surface contamination. As such, concentration and action times are usually much more than those obtained with suspension methods^67^. Therefore, the findings of this study can also be used for disinfecting suspensions as viruses in suspensions are easier to inactivate than viruses on surfaces^54^.

#### Effective exposure time for HCoV-229E disinfection

**Figure 6:**
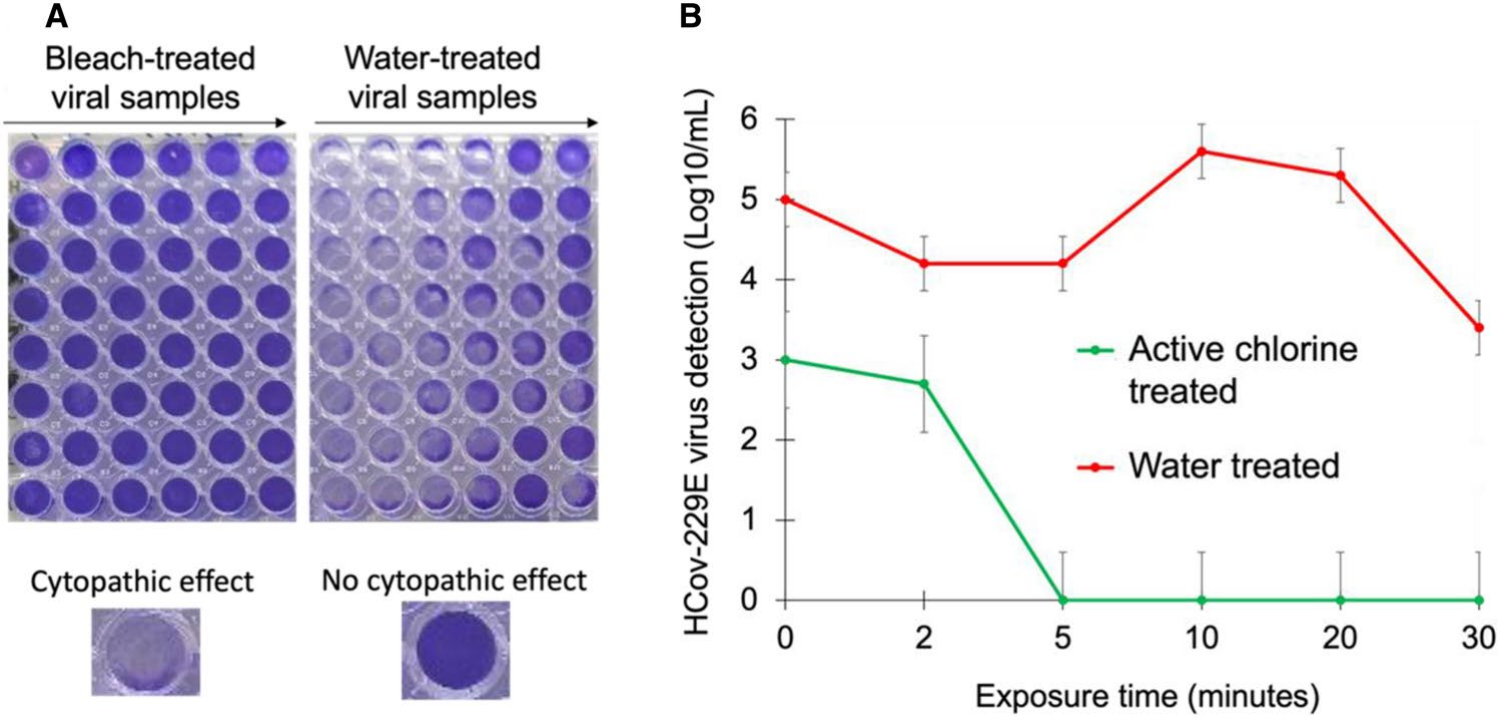
(**A**) Effect of exposure time on HCoV-229E inactivation by the Unipolar disinfectant vapour. The dilutions were from 10^–1^ to 10^–6^ from left to right for both treatments, (**B**) Effect of exposure time on HCoV-229E inactivation by the Unipolar disinfectant vapour. Note here that the *exposure time* is the time after the complete application of the disinfectant mist or water mist control. For this reason, some viral inactivation is observed at t = 0 for exposure time, due to some virus inactivation in the application phase of the experiment.

Next, the effect of exposure time on HCoV-229E virus inactivation was investigated. HCoV-229E samples were treated with active chlorine at a concentration of 400 ppm concentration and after application of the disinfectant mist, the samples were incubated further with exposure times of 0, 2, 5, 10, 20 and 30 min. All exposure times showed a decrease in TCID_50_, but a significant reduction was observed at exposure times 5 min and longer. This result indicates that active chlorine is effective in inactivating HCoV-229E with 400 ppm concentration within 5 min of exposure after application of the disinfectant. Figure 6A shows crystal violet stained Huh7 cell cultures in 96-well plates treated for 5 days with active chlorine-treated viruses (left 6 columns) and water-treated viruses (right 6 columns) using 5-min exposure times. High TCID_50_ units in log_10_/mL of HCoV-229E were recovered across all exposure periods from water-treated samples (Fig. 6B, red line). In contrast, a significant decrease in TCID_50_ units in log_10_/mL of HCoV-229E was observed in samples in which the virus was treated with the Unipolar-generated active chlorine (Fig. 6B, green line). The virus reduction after 5 min of exposure was at least 4.2 log_10_ compared to water-treated groups. On the contrary, samples treated with active chlorine at 0- and 2-min exposure showed less virus reduction (≤ 1.5 log_10_). These results can be seen in Fig. 6.

The results in Fig. 6 indicate that the minimum exposure time required for viral inactivation using the Unipolar ARD device is 5 min at its minimum effective concentration of active chlorine (400 ppm). It is important to note that any decrease in TCID_50_ units of HCoV-229E beyond 20 min exposure time might be partly due to the dryness effect on the viral sample over time, which is supported by a previous similar study^68^. It is notable that other studies have specified active chlorine concentrations of 2000 ppm or more are required to achieve significant viral load reductions in households and healthcare facilities^13,17^. However, more recent studies have demonstrated that 1000 ppm sodium hypochlorite concentration is effective in treating coronaviruses in only one minute^19^. The World Health Organisation also recommends the use of a chlorinated disinfectant concentration of at least 1000 ppm to be effective on surfaces within a few minutes^52^. The results for longer exposure time in this study are similar to the results of the previous studies and align with the World Health Organisation recommendations for liquid disinfectants, as using higher chlorinated disinfectant concentrations will decrease the exposure time required for successful inactivation of HCoV-229E^27,67^. This study achieves effective inactivation of HCoV-229E on a carrier at a significantly lower chlorinated disinfectant concentration of 400 ppm in only 5 min of exposure time. The efficacy of this method at lower concentrations and short exposure times is an advantage over previous studies, as it reduces the potential toxicity on humans and animals, conserves disinfectant, and enables longer operation time^52^.

It was also demonstrated in this study that the Huh7 cell cultures treated with active chlorine only (no virus) did not show cytopathic effects. This was true for all concentrations of chlorine disinfectant investigated. The absence of cytopathic effects suggests that the active chlorine concentrations used were not toxic to Huh7 cells. This is an important finding as cytotoxicity of chlorine bleach-based disinfectants has been observed at active chlorine concentrations of 800 ppm or higher at ≥ 2 min exposure times on human peripheral lymphocytes^69^, dermal fibroblasts^70^, ocular melanoma cells^71^, and osteoblasts^72^. More generally, toxicity to human cells is less of a concern for disinfection of unoccupied spaces, which is of relevance for decontamination of hospital wards, public facilities, shipping containers and quarantine areas.

## Conclusion

A rapid method for analysing the active chlorine concentration was established and used to optimize disinfectant vapour generated from the Unipolar ARD system. This disinfectant vapour was established as effective for inactivation of HCoV-229E virus on surfaces of carriers using low chlorine concentrations and short exposure times. With exposure times of 5 min at 400 ppm active chlorine, significant viral inactivation was observed, with no apparent toxic effect on Huh7 cells. This is the lowest effective chlorine disinfectant concentration reported in the literature for effective inactivation of coronavirus in short exposure times. The rapid, on-demand generation of disinfectants bodes well for virus inactivation on surfaces. Future studies will extend this evaluation to other viral and bacterial pathogens and biofilms, with potential applications for disinfection in public, domestic, healthcare, and industrial settings.

## Data Availability

The datasets generated and analysed during the current study available from the corresponding author on reasonable request.
